# High-fat diet impairs spatial memory and hippocampal intrinsic excitability and sex-dependently alters circulating insulin and hippocampal insulin sensitivity

**DOI:** 10.1186/s13293-016-0060-3

**Published:** 2016-01-28

**Authors:** Erica L. Underwood, Lucien T. Thompson

**Affiliations:** Cognition & Neuroscience Program, School of Behavioral & Brain Sciences, University of Texas at Dallas, 800 W. Campbell Rd., Richardson, TX 75080 USA

**Keywords:** Sex differences, Spatial memory, Hippocampal excitability, AHP, CA1, Glucose regulation, High-fat diet, Diabetes

## Abstract

**Background:**

High-fat diets promoting obesity/type-2 diabetes can impair physiology and cognitive performance, although sex-dependent comparisons of these impairments are rarely made. Transient reductions in Ca^2+^-dependent afterhyperpolarizations (AHPs) occur during memory consolidation, enhancing intrinsic excitability of hippocampal CA1 pyramidal neurons. In rats fed standard diets, insulin can enhance memory and reduce amplitude and duration of AHPs.

**Methods:**

Effects of chronic high-fat diet (HFD) on memory, circulating insulin, and neuronal physiology were compared between young adult male and female Long-Evans rats. Rats fed for 12 weeks (from weaning) a HFD or a control diet (CD) were then tested in vivo prior to in vitro recordings from CA1 pyramidal neurons.

**Results:**

The HFD significantly impaired spatial memory in both males and females. Significant sex differences occurred in circulating insulin and in the insulin sensitivity of hippocampal neurons. Circulating insulin significantly increased in HFD males but decreased in HFD females. While the HFD significantly reduced hippocampal intrinsic excitability in both sexes, CA1 neurons from HFD females remained insulin-sensitive but those from HFD males became insulin-insensitive.

**Conclusions:**

Findings consistent with these have been characterized previously in HFD or senescent males, but the effects observed here in young females are unique. Loss of CA1 neuronal excitability, and sex-dependent loss of insulin sensitivity, can have significant cognitive consequences, over both the short term and the life span. These findings highlight needs for more research into sex-dependent differences, relating systemic and neural plasticity mechanisms in metabolic disorders.

**Electronic supplementary material:**

The online version of this article (doi:10.1186/s13293-016-0060-3) contains supplementary material, which is available to authorized users.

## Background

Systematic studies of systemic and neuronal deficits linked to diet-induced glucose dysregulation are rare, while studies assessing them in reproductively normal females are nearly nonexistent. Recently, Underwood and Thompson [1] found that young adult male Long-Evans rats fed a high-fat diet (HFD) became obese and had elevated fasted blood glucose levels, elevated corticosterone, and impaired glucose tolerance, while females fed the same HFD exhibited only elevated corticosterone. However, regardless of peripheral metabolism alterations, both sexes fed the HFD were equally impaired in a spatial-object recognition memory task indicative of impaired hippocampal function. This study further elucidates the sexually dichotomous effects of a HFD on hippocampal function by examining dietary effects on cognitive performance, neuronal intrinsic excitability, and insulin sensitivity.

Insulin crosses the blood-brain barrier [2], activating neuronal insulin receptors (IRs) [3] which catalyze phosphorylation of IRS-1 and IRS-2. Insulin enhances NR-mediated synaptic plasticity in the CA1 of the hippocampus [4], a region necessary for many forms of learning and for memory consolidation [5]. Considerable evidence exists for insulin’s potential role in hippocampal memory: IR mRNA is highly expressed in the hippocampus [6], immediate post-acquisition infusion of insulin into the hippocampus enhances passive-avoidance memory in rats [7], and IR expression is up-regulated (and IR-insulin binding increased) in rat CA1 neurons after spatial learning [6, 8, 9].

Hippocampal pyramidal neurons exhibit prolonged afterhyperpolarizations (AHPs) following firing. AHPs clamp neuronal membrane potentials below firing thresholds, reducing intrinsic excitability and responses to afferents [10, 11]. In rats and rabbits, memory consolidation transiently reduces AHPs of hippocampal CA1 pyramidal neurons [12–16]. Additionally, while rapid modulation of AHPs by estradiol (with cognitive impact) has been demonstrated in neurons from female rats [17–21], systematic comparisons of AHPs in male and female neurons have not been made. As reported by Underwood and Thompson [1], circulating estradiol was unaltered by consumption of the HFD used in the current study and therefore an unlikely explanation for the effects seen.

Activity of both small-conductance Ca^2+^-activated (SK) K^+^ channels generating medium-duration AHPs (mAHPs) [22] and phosphorylation-sensitive slow AHPs (sAHPs), generated by ambiguously identified Ca^2+^-activated K^+^ channels [23–25], are transiently reduced after learning. Several studies have addressed the effects of glucose dysregulation and of insulin on AHPs and on intrinsic hippocampal excitability in male rats, but the results have been complex and sometimes contradictory, highlighting the importance of animal strain differences and timing of onset of high-fat diets. Pancani et al. [26] reported dietary effects on lipid metabolism but a lack of diet-dependent memory impairment; age-dependent deficits in spatial learning were associated with increases in CA1 mAHPs; and, most relevant for the current studies, significant reductions in CA1 sAHP insulin sensitivity were observed only in neurons from the control diet but not HFD-fed male rats. Chronic intraventricular perfusion of insulin into the brain of male control rats, mimicking the elevation seen in T2D insulin-dysregulation models, has been shown to increase sAHPs [27]. An earlier report [28] also found enhanced sAHPs in CA1 neurons from streptozotocin-induced male diabetic rats. Maimaiti et al. [29] found that low-dose administration of intranasal insulin improved memory in aged animals and ex vivo insulin reduced AHPs, consistent with findings that AHP reductions are a mechanism for improved memory [12–16].

Our experiments further extend these findings, while addressing sex as a critical variable. Our findings demonstrate major sex-dependent differences in systemic and neuronal responses to a high-fat diet, as well as sex differences in insulin sensitivity of calcium-dependent AHPs in CA1 pyramidal neurons.

## Methods

### Subjects

Throughout the study, young Long-Evans outbred rats were socially housed on a 12-h light/dark cycle with ad libitum access to food and water according to their assigned diet and were classed as young adults at the time of all testing. Feeding (detailed below) began at weaning in matched cohorts of males and females from the same litters. Daily records of weight were maintained throughout the study. All procedures were conducted in accordance with the current Institutional Animal Care and Use Committee regulations of The University of Texas at Dallas as well as all guidelines of the USDA. All rats were well handled for 10 min daily for 5–7 days prior to all testing procedures to minimize stress and variability due to extraneous factors. Vaginal cytology samples were collected on all females either immediately after behavioral training or under anesthesia prior to tissue collection in order to assess the estrous cycle phase at the time of experimentation. Animals were classified as being in estrous, diestrous, or proestrous based on the presence of cornified epithelial cells, nucleated epithelial cells, or leukocytes, respectively (as described by Marcondes et al. [30]).

### Diet

All subjects were fed their assigned diet (from weaning) for 12 ± 1.3 weeks prior to behavioral and physiological assessment. Control diet (CD) groups received 14 % fat, 64.8 % carbohydrate, and 21.2 % protein rat chow (Open Source Diets) along with pure filtered water. High-fat diet (HFD) groups received 58 % fat, 25.5 % carbohydrate, and 16.4 % protein rat chow (Open Source Diets chow was augmented with additional saturated fat (coconut oil, the primary source of dietary lipids in the control diet) and casein protein (the primary source of dietary proteins in the control diet) to achieve desired dietary ratios). Nutritional sufficiency was confirmed by analysis by Open Sources Diets.

### Spontaneous alternation task

**Fig. 1:**
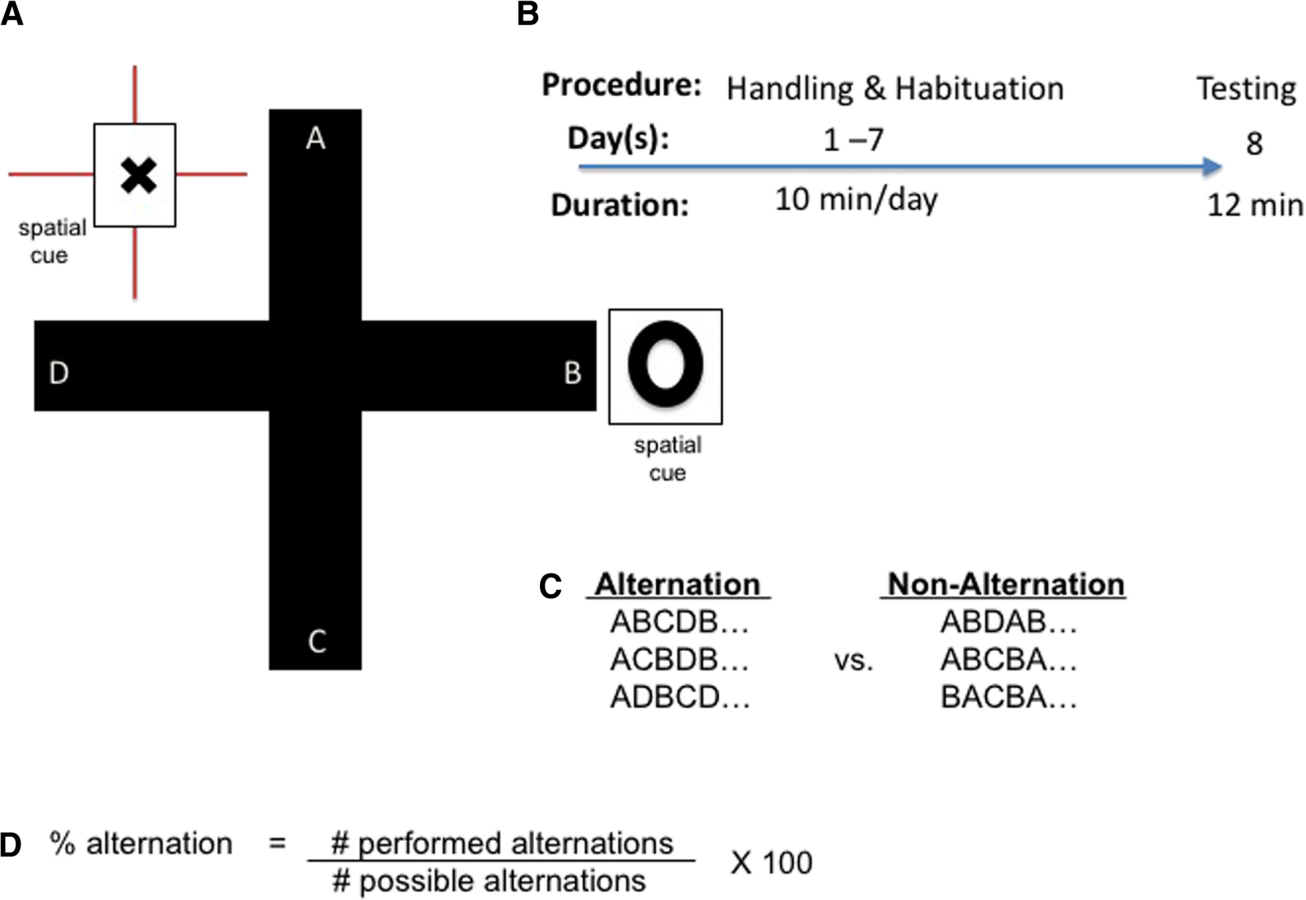
The spontaneous alternation task used to assess spatial memory. **a** A four-arm radial plus maze, with two explicit distal visual cues for spatial orientation. No explicit rewards were present on the maze. **b** The time line used to ensure the behavior of all rats was minimally impacted by stressors related to the environment or the experimenters. **c** Parsing of exploratory behavior into sets of alternations or non-alternations. **d** Conversion of raw behavioral observations into % alternation scores for comparisons between individuals and between groups

Rats were tested for performance on a spontaneous alternation task in a four-arm radial maze (plus maze). The plus maze consisted of four arms joined to a central 20 × 20 cm platform. Each arm was 58 cm long and 13 cm wide, with 24-cm walls. Rats were handled and allowed to acclimate to the testing room for 10 min a day for 7 days prior to testing. Spontaneous alternation testing was performed by placing the rat in the center area of the maze (all animals started facing the same direction) and allowing 12 min of free exploration [31]. Arm entries were monitored and recorded remotely via live video feed; an alternation was defined as four different arm choices out of five consecutive arm entries. An alternation score was calculated by dividing the number of alternations (in overlapping quintuplets of trials) by the number of possible alternations and multiplying by 100 (Fig. 1).

### Brain slice preparation

In order to isolate the effects of the HFD from potential behavioral training effects on neuronal intrinsic excitability, a separate group of behaviorally naive rats were anesthetized with ~2 % isoflurane and rapidly decapitated. To avoid stress-related confounds on electrophysiological measures, subjects were not fasted prior to slice preparation. Trunk blood was collected and centrifuged (3800×*g*) in heparinized saline for 10 min, after which plasma samples were pipetted off and stored frozen for later ELISA assays (see below). Brains were rapidly removed (within 30 s of decapitation) and immersed in ice-cold (0–1 °C) oxygenated (95 % O_2_–5 % CO_2_) sucrose artificial cerebrospinal fluid (aCSF), with an equimolar concentration of sucrose to replace NaCl containing (in mM) the following: 3.0 KCl, 1.3 MgSO_4_, 1.24 NaH_2_PO_4_·H_2_0, CaCl_2_·2H_2_O, 220 sucrose, 26.0 NaHCO_3_, and 10.0 d-glucose; pH 7.4. Hippocampal sections (400 μm) were cut using a vibratome. Sections were then transferred to a holding chamber containing room temperature (~23 °C) oxygenated normal aCSF containing (in mM) the following: 3.0 KCl, 1.3 MgSO_4_, 1.24 NaH_2_PO_4_·H_2_0, CaCl_2_·2H_2_O, 124 NaCl, 26.0 NaHCO_3_, and 10.0 d-glucose; pH 7.4. Sections were incubated in the holding chamber for at least 1 h, after which sections were transferred, one at a time, to a recording chamber and perfused with normal aCSF at 31 °C ± 1 °C for intracellular recording.

### Brain slice neurophysiological recordings

Recording electrodes were pulled from thick-wall (O.D. 1.5 mm, I.D. 0.89 mm) borosilicate capillary tubing. Electrodes were filled with 3 M KCl, and those with a series resistance of 30–80 MΩ used for recording. Current and voltage traces were collected at 10 KHz (National Inst.). A series of 400-ms current pulses ranging from −1.0 to +0.2 nA were injected into the neuron, and the subsequent voltage responses recorded using an AxoClamp 2B (Axon Inst.) amplifier for current-voltage analyses. Input resistance was calculated as the slope of the linear regression line produced from the current-voltage curve between −0.6 and −0.05 nA. Hyperpolarizing sag was calculated as the difference between the maximum negative membrane potential during the first 100 ms and the average membrane potential during the last 75 ms of −1.0-nA 400-ms stimulus pulses and was used as an indirect measure of *I*_H_ [32], another conductance shown to be transiently increased in some forms of learning in the CA1 or in the layer V prefrontal cortical but not in CA3 pyramidal neurons [15, 33, 34]. A 100-ms positive current pulse, sufficient to elicit four action potentials during the current pulse, was used to evoke post-burst afterhyperpolarizations (AHPs). The peak AHP was calculated as the difference between the resting membrane and the peak negative membrane potential after the termination of the stimulus pulse, another measure of the AHP known to be modulated transiently after learning new tasks [13, 35–37].

AHP durations were calculated as the time required for AHPs to return to the resting membrane potential for at least 10 ms after the termination of stimulus pulses. AHP amplitudes were assessed at specific intervals following the stimulus to assess both medium (mAHP, 250 ms post-burst) and slow (sAHP, 500–4000 ms post-burst) components of the AHP [13, 37–42]. Accommodation measures were calculated as the number of spikes elicited during 800-ms stimulus pulses of the same amplitude used to evoke four-spike AHPs. Resting membrane potentials were calculated as the difference between the potential before and after withdrawal of recording electrodes from neurons. Inclusion criterion for healthy neurons included a resting membrane potential of −68 ± 4 mV, action potential amplitude ≥80 mV, and input resistance ≥30 MΩ.

### Insulin sensitivity via bath application

After initial recordings were taken in normal aCSF perfusion, a second perfusion of insulin-treated aCSF was initiated to assess effects of a bath application of insulin on excitability measures in the hippocampus. (Dose-response studies were first performed (see Additional file 1) to determine the optimal dose of insulin to use to remain within stable physiological parameters.) Insulin was allowed to perfuse the slice for a minimum of 8 min before recordings were initiated again in order to investigate insulin’s sex-dependent effects and the effects of diet on insulin sensitivity. The insulin concentration tested was 12.5 nM, as determined via a dose-response study prior to experimentation (Additional file 1: Figure S1).

### Plasma insulin analysis

Non-fasted plasma samples were aliquoted into 500-μL tubes and frozen until use to avoid repeat freeze/thaw cycles. The plasma was thawed at room temperature for 1 h and then diluted appropriately for ELISA assays. Insulin was assessed using rat insulin ELISA kits (CrystalChem) using an ELx800 plate reader (BioTek) and Gen5 software.

### Analyses

Raw neurophysiological data was sampled and stored digitally using LabView software (National Inst.) and Igor (WaveMetrics). Statistical analyses were run using StatView (SAS Inst.) for neurophysiological measures and Prism 6 (GraphPad) for behavioral and ELISA measures. Significant differences between sexes and dietary conditions were assessed using planned two-way ANOVAs and Tukey’s post hoc tests for multiple comparisons.

## Results

A total of 38 young-adult male rats fed the control diet and 30 young-adult male rats fed the HFD from weaning along with 21 young-adult female rats fed the control diet and 29 young-adult female rats fed the HFD from weaning were used to generate the data presented here. All diet- and sex-dependent effects were assessed in matched littermate cohorts (i.e., both males and females from the same litters were assigned to each treatment condition), with results reported as means ± SEM. Scatterplots of the raw behavior data are included for emphasis.

1. High-fat diet impairs spatial memory in both sexes.
  A total of 30 rats were used to assess spatial memory performance on the spontaneous alternation task. No sex differences in performance were seen in comparisons of either CD-fed animals (*p* > 0.8) or HFD-fed animals (*p* > 0.8). However, both male and female rats fed the HFD had significantly lower alternation scores relative to their CD counterparts (*F* (3,30) = 8.12, *p* = 0.0004; Fig. 2a). Males fed the HFD exhibited a 14.3 % reduction in spontaneous alternation compared to CD-fed males (*p* = 0.0197) while HFD females exhibited a 13.7 % reduction in spatial memory performance (*p* = 0.0013). These profound deficits in spatial memory performance in HFD cohorts are consistent with and corroborate the earlier report from our laboratory [1] of deficits in hippocampal-dependent spatial memory in comparisons of the performance of male and female rats fed the same HFD or CD in a spatial-object recognition task. The spatial memory impairment was not due to reductions in motor activity, as evidenced by equivalent number of arm entries made in the plus maze during the exploratory behavior scored for alternation (*p* > 0.2; Fig. 2b).

**Fig. 2:**
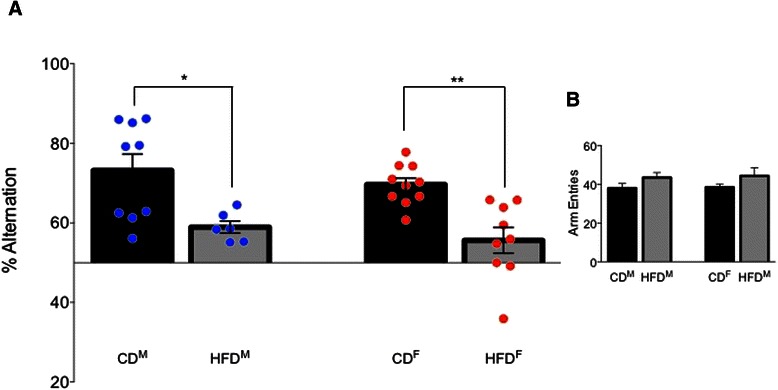
HFD impaired spatial memory on **a** the spontaneous alternation task in both males and females but did not significantly alter total exploration **b** of the plus maze (*p* > 0.2). *p < 0.05; **p < 0.01
2. Sex-dependent hippocampal CA1-neuron intrinsic excitability.
  A total of 23 CA1 pyramidal neurons from male rats fed the control diet were studied and intracellular recording measures from these neurons compared to those from 14 CA1 pyramidal neurons from female rats fed the control diet. Average AHP and accommodation traces are shown from male CA1 neurons (see Figs. 3a and 4a) and from female CA1 neurons (see Figs. 3d and 4c). Table 1 lists biophysical and physiological measures that did not differ significantly between neurons from male or female rats.

**Fig. 3:**
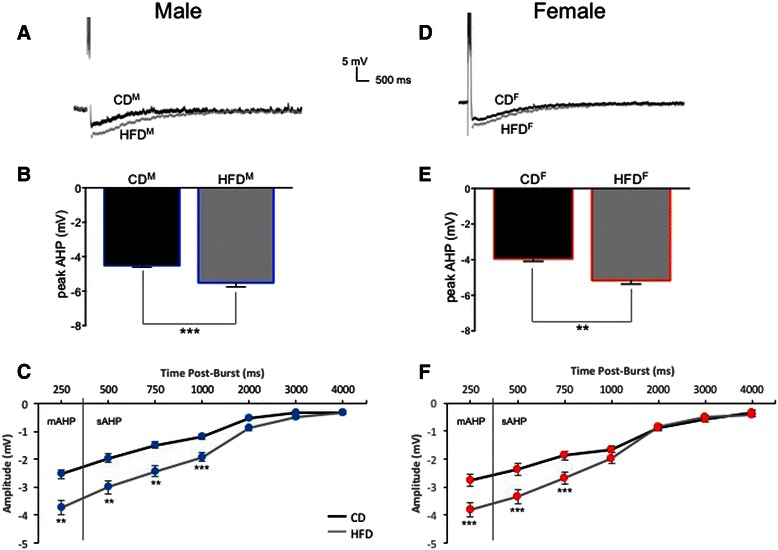
Ventral CA1 pyramidal neurons showed sex- and diet-dependent differences in intrinsic excitability, here assessed as differences in post-burst AHPs (average traces from male (**a**) and female (**d**) neurons are shown). The HFD enhanced peak AHP amplitudes significantly in both males (**b**) and females (**e**), but did not significantly increase AHP duration in either sex. No sex differences were observed in measures of medium or slow AHPs from CA1 neurons of control-fed males (**c**) or females (**f**). The HFD significantly enhanced both mAHP and sAHP measures in males (**c**) and more profoundly enhanced mAHP and sAHP components in female neurons (**f**). **p < 0.01; ***p < 0.001

**Fig. 4:**
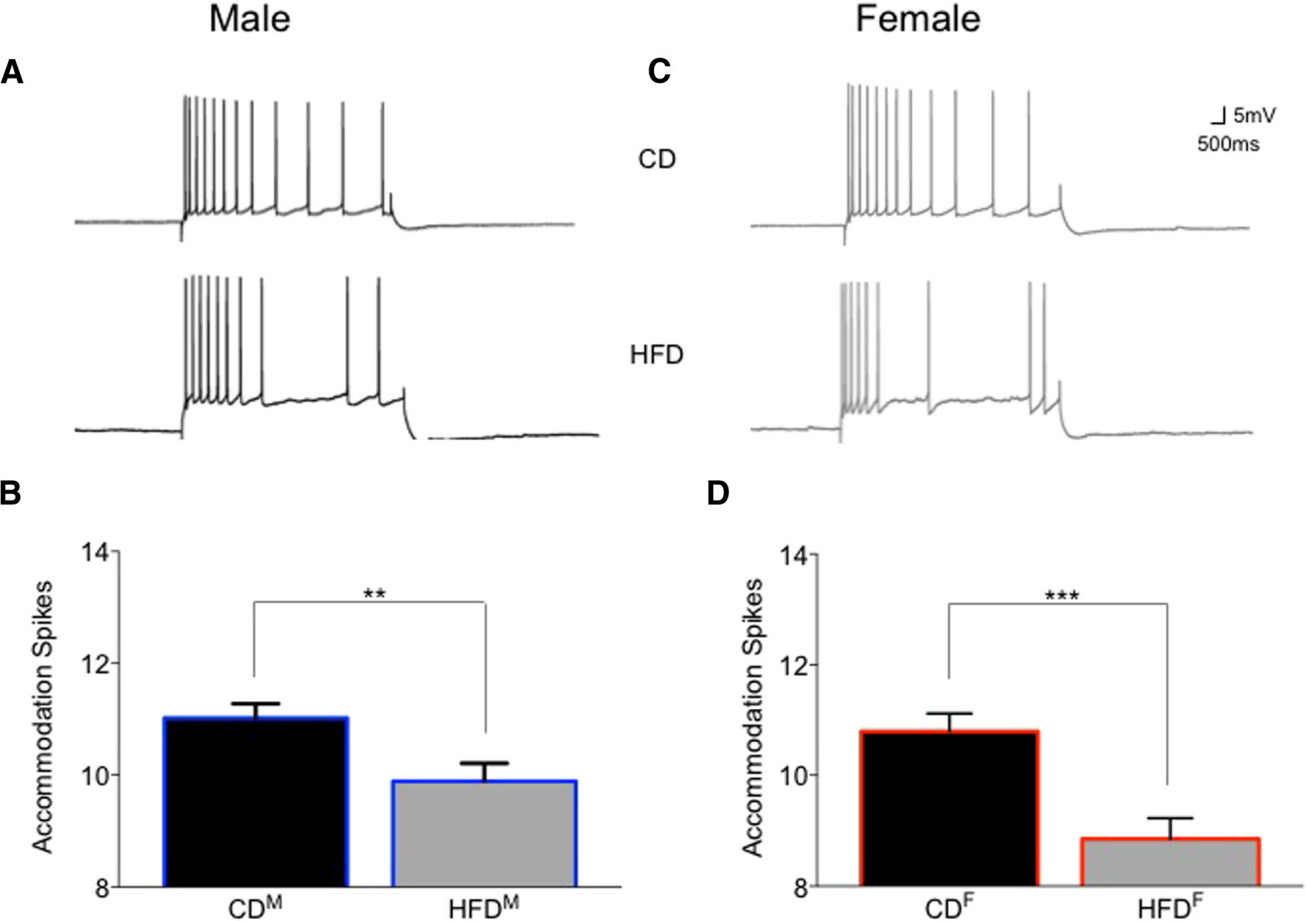
Further evidence of diet-induced reductions in intrinsic excitability is seen in measures of spike-frequency accommodation of ventral CA1 pyramidal neurons. Representative examples of accommodation of male neurons (**a**) and female neurons (**c**) from rats fed the two diets for 15 weeks are shown. Significantly increased accommodation (i.e., decreased action potential firing) to a sustained depolarizing pulse was observed in both male (**b**) and female (**d**) HFD CA1 neurons. **p < 0.01; ***p < 0.001

**Table 1:** Biophysical measures in CA1 pyramidal neurons that were not altered by the high-fat diet (HFD) in young adult male and female rats

	Rats (*n*)	Neurons (*n*)	Input resistance (MΩ)	Sag (mV)	Resting potential (mV)
Control male					
aCSF	22	23	81.0 ± 2.4	8.3 ± 0.6	−70.2 ± 0.3
Insulin		15	85.9 ± 3.0	10.2 ± 0.1	−66.0 ± 1.3
HFD male					
aCSF	14	24	66.7 ± 2.3	8.1 ± 0.6	−67.5 ± 0.6
Insulin		13	64.3 ± 3.5	8.9 ± 1.0	−68.7 ± 0.4
Control female					
aCSF	7	14	59.6 ± 3.4	4.6 ± 0.8	−71.6 ± 0.4
Insulin		8	53.6 ± 1.2	7.1 ± 0.9	−66.7 ± 0.6
HFD female					
aCSF	14	20	85.5 ± 4.8	6.5 ± 0.5	−70.3 ± 0.4
Insulin		9	87.0 ± 6.9	10.1 ± 2.2	−63.3 ± 1.7
  Post-burst AHPs from control CA1 neurons exhibited larger peak amplitudes in slices from male rats than from females (−4.47 ± 0.11 mV (Fig. 3b) vs. −3.98 ± 0.11 mV (Fig. 3e); *F* (1,36) = 10.40, *p* = 0.01). However, AHP duration was significantly longer in neurons from control females than males (3606.5 ± 303.2 ms vs. 2524.5 ± 265.0 ms; *F* (1,36) = 7.07, *p* = 0.01). No significant differences were observed in comparisons of mAHPs (*p* = 0.7) or in amplitude measures of the sAHP (*p* = 0.3) between neurons from control-fed male (Fig. 3c) or female (Fig. 3f) rats.
  Another measure of intrinsic excitability, spike-frequency adaptation or accommodation to a sustained depolarization, did not differ significantly between the sexes in CA1 pyramidal neurons from control-fed male or female rats (Fig. 4b, d, *p* = 0.98).
  It should be noted that the variance (here expressed as standard errors) was not elevated in biophysical measures obtained from CA1 neurons from females when contrasted with that obtained from males.
3. Sex-dependent diet-induced reduction in hippocampal intrinsic excitability.
  CA1 pyramidal neurons from male rats fed the HFD (*n* = 24) were less excitable than those from males fed the CD (*n* = 23; see Fig. 3a). Post-burst AHPs of neurons from male HFD-fed rats exhibited significantly larger peak amplitudes than those from male CD-fed rats (*F* (1,46) = 6.85, *p* = 0.0001, Fig. 3b). No significant differences were observed in AHP duration or in AHP area from CA1 neurons comparing measures from HFD- to CD-fed male rats (*p* = 0.11). However, significant differences were observed in medium (*F* (1,46) = 9.67, *p* = 0.002) and in multiple slow components of the AHP (500 ms post-burst, *F* (1,46) = 8.48, *p* = 0.004; 750 ms post-burst, *F* (1,46) = 10.47, *p* = 0.001; and 1000 ms post-burst, *F* (1,46) = 8.40, *p* = 0.004) when comparing data from neurons of HFD- to CD-fed male rats (Fig. 3c). Biophysical measures that did not differ significantly between neurons from males fed the CD and those fed the HFD are shown in Table 1.
  Post-burst AHPs of neurons from female rats fed the HFD (*n* = 20) exhibited significantly larger peak amplitudes than those from female rats fed the CD (*n* = 14; *F* (1,33) = 21.27, *p* = 0.0001; see Fig. 3e). No significant differences were observed in AHP duration or in AHP area from CA1 neurons comparing between HFD- or CD-fed female rats (*p* = 0.12). However, significant differences were observed in medium (*F* (1,33) = 13.99, *p* = 0.0002) and slow components of the AHP (500 ms post-burst, *F* (1,33) = 10.85, *p* = 0.001; 750 ms post-burst, *F* (1,33) = 11.59, *p* = 0.0008) when comparing between neurons from HFD- and CD-fed female rats (Fig. 3f). Biophysical measures that did not differ significantly between neurons from females fed the CD and those fed the HFD are shown in Table 1.
  Accommodation, another measure of intrinsic excitability, was also significantly altered by diet in young adult rats (*F* (3,46) = 8.96, *p* < 0.0001), with sex-dependent differences in the magnitude of diet-induced enhancement of accommodation (reduction of excitability) observed (see Fig. 4). Neurons from male HFD rats exhibited significantly enhanced accommodation, firing fewer action potentials (APs) per burst in response to a sustained depolarization, than neurons from male CD rats (a decrease of 1.4 APs for the HFD; *p* = 0.0092). This diet-dependent effect was slightly more pronounced in females; neurons from female HFD rats exhibited significantly enhanced accommodation, firing fewer action potentials per burst in response to a sustained depolarization, than neurons from female CD rats (a decrease of 2 APs for the HFD; *p* = 0.0009). Notably, enhanced accommodation has been reported previously in senescent memory-impaired animals [43–47].
4. Sex- and diet-dependent effects on circulating insulin.
  Resting serum insulin concentrations varied significantly between sexes and diet treatments (see Fig. 5; *F* (3,35) = 5.11, *p* = 0.005). Although serum insulin concentrations in control diet rats were higher in females compared to males, the difference was not statistically significant (*p* = 0.2). Along with our previous observation of increased fasting blood glucose in HFD-fed males [1], serum insulin concentrations were also significantly elevated in HFD-fed males compared to CD-fed males (*p* = 0.02), both clinical indicators of type-2 diabetes [48]. Surprisingly, instead of the diabetes-like elevation seen in HFD males, serum insulin concentrations were significantly reduced in HFD-fed females compared to CD females (*p* = 0.005). These findings are consistent with Mehran et al. [49], where female mice were found to be resistant to HFD-induced hyperinsulinemia and to HFD-induced obesity. It is important to note, however, that the mice used in that study were deficient in Ins2 gene expression, the source of brain-expressed insulin, whereas our subjects are an outbred strain. Taken together, these studies both provide evidence for a sexually dichotomous role of insulin in the development of type-2 diabetes. Future work in our own laboratory plans to more fully address sex-dependent differences in insulin signaling and glucose regulation in the brain, including endogenous production of insulin in the brain in our HFD rat model.

**Fig. 5:**
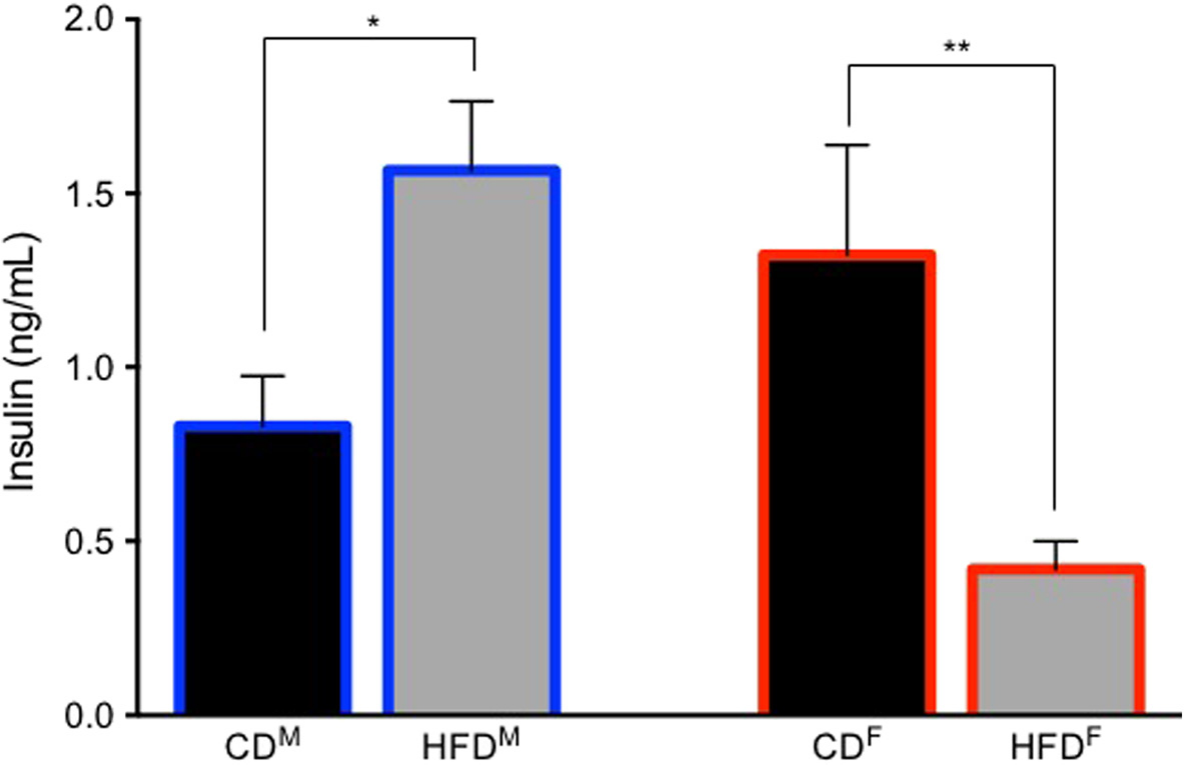
Sex-dependent effects of the HFD on circulating insulin. In males, the HFD increased circulating insulin compared to controls. However, in females, the HFD significantly decreased circulating insulin compared to controls. While this increased insulin in HFD-fed male rats met a clinical criterion for type-2 diabetes, HFD-fed females not only did not exhibit this clinically relevant profile, but in fact exhibited anomalous decreases in circulating insulin. *p < 0.05; **p < 0.01
5. Sex- and diet-dependence of hippocampal CA1-neuron insulin sensitivity.
  As reported in (3) above, CA1 pyramidal neurons from both male and female HFD-fed rats were less intrinsically excitable than those from their CD-fed littermates when recorded in normal aCSF (Figs. 3 and 4). Given the changes in circulating insulin seen above (Fig. 5), insulin sensitivity of CA1 neurons was next assessed. Consumption of the HFD abolished sensitivity of the AHP (on multiple measures) to bath-applied insulin in male neurons (Fig. 6). CA1 neurons from female HFD rats, however, not only remained insulin-sensitive, but in fact insulin sensitivity was enhanced in these neurons compared to those from female CD rats (Fig. 7).

**Fig. 6:**
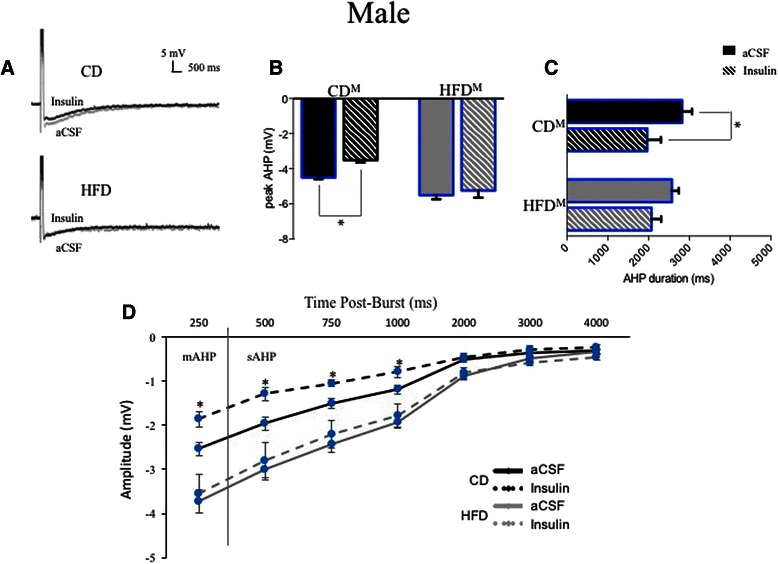
Sex- and diet-dependent responses to bath application of 12.5 nM insulin in CA1 pyramidal neurons from male rats. **a** Averaged AHP traces are shown from CD (insulin-sensitive) neurons and HFD (insulin-insensitive) male neurons. **b** Bath application of insulin significantly reduced peak AHP amplitudes in CD but not HFD neurons. **c** AHP durations were significantly reduced in CD neurons, but not in HFD neurons. **d** While mAHP and sAHP measures were significantly reduced by insulin in neurons from CD rats, both mAHPs and sAHPs from HFD rats were insulin-insensitive. *p < 0.05

**Fig. 7:**
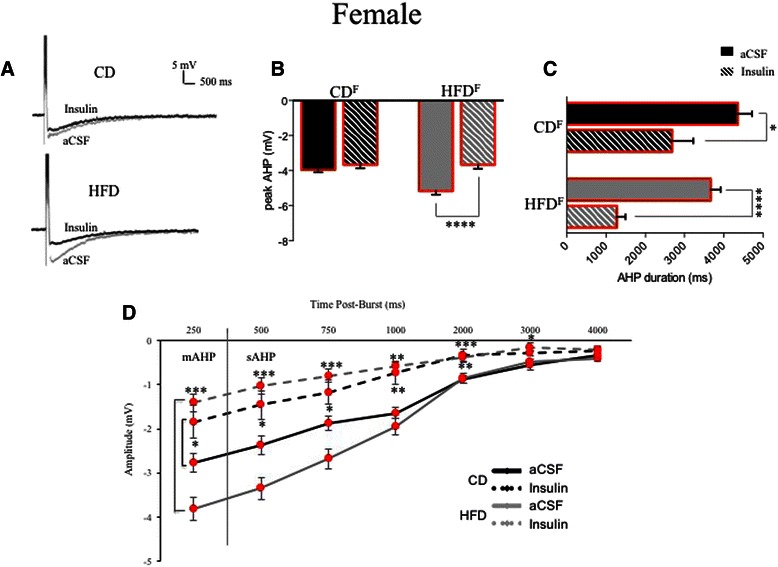
Diet-dependent responses to bath application of 12.5 nM insulin in CA1 pyramidal neurons from female rats. **a** Averaged AHP traces are shown from both CD neurons and HFD neurons. **b** Bath application of insulin had no effect on peak AHP amplitudes in CD neurons but significantly reduced peak AHPs in HFD neurons. **c** While AHP durations were significantly reduced by insulin in CD neurons, significantly larger reductions were observed in HFD neurons. **d** Both mAHP and sAHP measures were significantly reduced by insulin in neurons from CD rats (*black bars at left* indicate comparisons made). Notably, insulin produced a more effective reduction of mAHPs and sAHPs in neurons from HFD rats (*gray bars at left* indicate comparisons made), continuing much longer post-burst, i.e., insulin significantly enhanced intrinsic excitability for many seconds in neurons from female HFD rats. Unlike in males, where HFD neurons lost insulin sensitivity on multiple measures, insulin sensitivity of AHPs actually increased in neurons from HFD female rats. *p < 0.05; **p < 0.01; ***p < 0.001; ****p < 0.0001
  Bath application of 12.5 nM insulin (a concentration previously determined to be effective in a dose-response study; Additional file 1: Figure S1) significantly reduced AHP peak amplitudes in neurons from male rats fed the CD (*n* = 15; Fig. 6b, *F* (3,27) = 12.23, *p* < 0.0001) but not in neurons from male rats fed the HFD (*n* = 13; *p* = 0.94). Insulin also significantly reduced mAHPs in neurons from male rats fed the CD (Fig. 6d, *F* (1,27) = 3.84, *p* = 0.0156), while mAHPs in neurons from male rats fed the HFD were insulin-insensitive (*p* = 0.92). Insulin significantly reduced several measures of the sAHP in neurons from male rats fed the CD, but sAHPs in neurons from male rats fed the HFD were insulin-insensitive (see Fig. 6d). In neurons from male CD-fed rats, insulin significantly reduced sAHPs (increasing CA1 pyramidal neuron intrinsic excitability) 500 ms (*F* (3,27) = 5.88, *p* = 0.012), 750 ms (*F* (3,27) = 3.75, *p* = 0.026), and 1000 ms post-burst (*F* (3,27) = 3.69, *p* = 0.031), while sAHPs were insulin-insensitive in neurons from male HFD-fed rats across all intervals tested (*p* = 0.63). Insulin significantly reduced AHP duration in neurons from CD- (*F* (3,27) = 3.498, *p* = 0.028) but not HFD-fed (Fig. 6c, *p* = 0.53) male rats.
  Unlike the insulin insensitivity seen in HFD males, insulin significantly reduced AHP peak amplitudes in neurons from female rats fed the HFD (*n* = 9; Fig. 7b, *F* (3,21) = 4.30, *p* = 0.04) but not in neurons from female rats fed the CD (*n* = 8; *p* = 0.21). Application of 12.5 nM insulin also significantly reduced measures of the mAHP in neurons from female rats fed the HFD (*F* (3,28) = 13.70, *p* = 0.0003), more so than in neurons from female rats fed the control diet (Fig. 7d, *F* (3,21) = 3.88, *p* = 0.05). Unlike in males, bath application of 12.5 nM insulin significantly reduced measures of the sAHP in neurons from female rats fed both the HFD and the control diet (see Fig. 7d). Measures of sAHPs were significantly reduced 500 ms post-burst (*F* (3,28) = 14.04, *p* = 0.0003), 750 ms post-burst (*F* (3,28) = 14.76, *p* = 0.0002), and 1000 ms post-burst (*F* (3,28) = 13.17, *p* = 0.0004), as well as 2000 ms post-burst (*F* (3,28) = 7.20, *p* = 0.008), 3000 ms post-burst (*F* (3,28) = 10.41, *p* = 0.002), and 4000 ms post-burst (*F* (3,28) = 5.40, *p* = 0.02) in neurons from female HFD-fed rats. While mAHPs in CA1 neurons from control-fed females were insulin-sensitive (Fig. 7d; *F* (3,21) = 5.47, *p* = 0.02), sAHP measures in CA1 neurons from control-fed females were insulin-sensitive only at intervals up to 1 s post-burst (Fig. 7d; 500 ms (*F* (3,21) = 4.73, *p* = 0.03); 750 ms (*F* (3,21) = 5.78, *p* = 0.02); 1000 ms (*F* (3,21) = 9.75, *p* = 0.003)). Unlike in males, significant reductions were observed in total AHP duration (Fig. 7c, *F* (3, 21) = 3.94, *p* = 0.01) after bath application of insulin to female CA1 neurons from control-fed rats. AHP durations were still more significantly reduced after bath application of 12.5 nM insulin to neurons from female HFD-fed rats (*F* (3,21) = 3.50, *p* = 0.0001).
  Accommodation, another measure of intrinsic excitability, was also significantly altered by bath application of insulin in young adult rats (*F* (7,48) = 7.719, *p* = 0.0001), with sex- and diet-dependent differences in the magnitude of the effects observed (see Fig. 8). As noted in (3) above, CA1 neurons from HFD males and females exhibited significantly enhanced accommodation compared to neurons from CD males or females. The accommodation of CA1 pyramidal neurons from both male (Fig. 8a; *p* > 0.3) and female (Fig. 8c; *p* > 0.9) CD rats was unchanged by bath application of insulin. However, effects of bath-applied insulin on accommodation of CA1 pyramidal neurons from HFD rats differed by sex. Consistent with the insulin insensitivity seen in the AHPs, the accommodation of neurons from HFD male rats was also not altered by bath-applied insulin (Fig. 8b; *p* > 0.9). However, not only was accommodation reduced by insulin in CA1 pyramidal neurons from female HFD rats (*p* < 0.0001), these neurons actually became more excitable than neurons from CD rats in normal aCSF (firing 12.25 APs vs. 10.8 APs respectively; Fig. 8d), again indicating a profound sexual dichotomy in insulin sensitivity of excitability measures between neurons from HFD-fed animals.

**Fig. 8:**
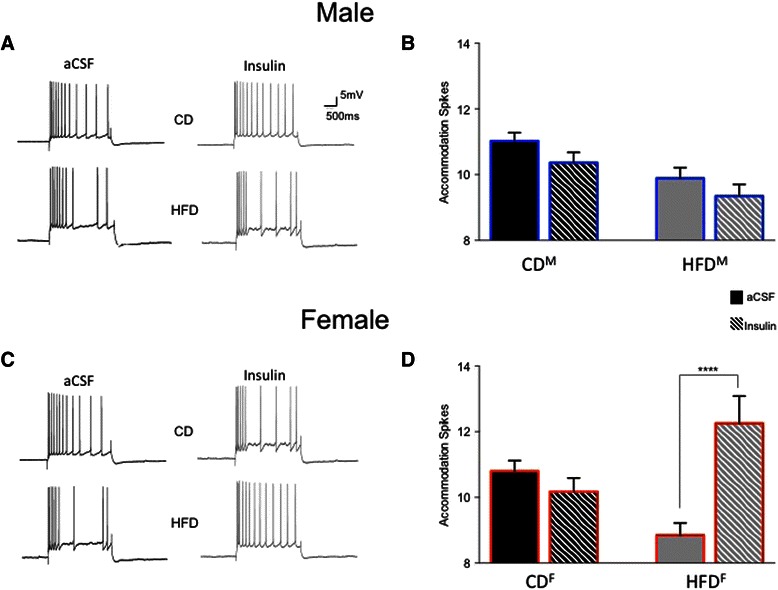
Sex- and diet-dependent effects of insulin were observed in measures of spike frequency accommodation. In neurons from CD **a** males and **c** females, the bath application of insulin did not alter accommodation, with no significant change in firing seen (**b**, **d**). Accommodation of HFD male neurons (**a**) was also unaffected, with no significant change in firing in the presence of insulin (**b**). In neurons from HFD females, the bath application of insulin (**c**) actually reduced accommodation, with a significant increase in action potential firing to a sustained depolarizing pulse observed (**d**). Again, while the HFD did not alter insulin sensitivity in males, it enhanced insulin sensitivity in females. ****p < 0.0001

## Discussion

Previously, we found sex differences for the HFD on weight gain, fasted circulating glucose, circulating cortisol, and glucose- and insulin-tolerance testing, with dysfunctional glucose regulation in male but not female HFD rats [1]. This study expands upon those initial findings, adding additional behavioral and biophysical data from CA1 neurons to account for some of the sex- and diet-dependent differences. Male HFD rats exhibit all clinical characteristics of type-2 diabetes seen in other diet-induced rodent T2D models [26, 50, 51], including increases in circulating insulin (Fig. 5). However, this clinically relevant profile is not seen in female HFD rats, and the significant decrease in circulating insulin in HFD females reported here indicates that compensatory changes to the diet occur in females. Additional work is required to explain these compensatory mechanisms and to more fully account for the range of sex differences observed. The range of pathological changes (behavioral and physiological) observed in this HFD model along with the rapid global increase in the incidence of type-2 diabetes in human children and young adults [48, 52–58] are an imperative for further study in both females *and* males.

In the present study, we found additional significant impairments in spatial memory (Fig. 2) and significant HFD-induced impairments in basal intrinsic excitability (Figs. 3 and 4) of hippocampal CA1 pyramidal neurons from both males and females, a profile typically only seen in senescent memory-impaired rats [44, 45, 59–61]. Male neurons exhibited diet-induced loss of AHP insulin sensitivity (Figs. 6 and 8), impacting all components of the AHP, i.e., a broader effect on Ca^2+^-dependent K^+^ channels than the loss of insulin sensitivity restricted to the sAHP reported by Pancani et al. [26]. Enhanced hippocampal insulin sensitivity observed in neurons from HFD females supports the hypothesis of profound sex differences within brain insulin-signaling pathways. In humans, acute intranasal insulin administration has been shown to reduce food intake in men, but not in women, whereas it improves spatial memory function in women, but not men [62]. There are likely sex differences not only in the insulin-signaling pathway but also in the functional salience of insulin between males and females.

Notably, the HFD did not alter circulating estradiol in young female rats [1], so estrogenic effects cannot account for the loss of intrinsic excitability in neurons from HFD female rats studied here. We also examined estrous staging of all female rats whose CA1 neurons were studied, and no cycle-dependence was observed (data not shown). The variance in measures of intrinsic excitability from female CA1 neurons was consistently comparable to that of males, again strongly arguing for the inclusion of females in future studies of hippocampal intrinsic excitability.

Our findings can be contrasted with those of Hwang et al. [63], who found LTP was impaired in male mice fed a HFD compared to females on the same diet. They found CD female mice had lower basal circulating insulin than males, i.e., baseline metabolic differences, an effect not observed in our rats. Since Hwang et al. stated that fully 50 % of their female mice were also acyclic, variable estrogen was an additional potential confound in their results. In the CA1 of the hippocampus, estrogen is known to enhance excitatory synaptic transmission and improve working memory [64, 65]. Much of the published physiological research with female rats involves ovariectomized subjects. Ovarian hormone loss results in rapid progressive loss of CA3–CA1 synaptic transmission [66], so estradiol administration is a common corrective. Unfortunately, despite noncyclic estradiol treatment, many studies report timing-, concentration-, and/or subject-dependent effects [67], i.e., increased variability in females that does not improve experimental clarity. Biophysical measures of neuronal function remain grossly understudied in reproductively intact females, with a dearth of direct comparisons to males [68, 69].

In our experiments, measures of intrinsic excitability of CA1 pyramidal neurons from young female CD rats were comparable to those obtained from their male counterparts; however, CA1 neurons from female HFD rats remained insulin-sensitive, while those from males lost insulin sensitivity. Sexually dichotomous effects of the HFD on circulating insulin, which significantly increased in males but decreased in females fed the HFD, may contribute to the observed divergent neuronal insulin sensitivity and require additional experimental investigation. Future clinical or experimental studies using exogenous insulin supplementation, e.g., modeled on findings in [29, 70] or [71], should carefully compare response measures between males and females. While systemic changes in glucose regulation have been reasonably well characterized in male models of type-2 diabetes, they are only infrequently studied in female model systems. Our findings of dietary impairment of memory, and the related loss of intrinsic excitability of young hippocampal neurons with sex differences in hippocampal insulin sensitivity, are unique.

## Conclusions

Our investigations of sex-dependent effects of a high-fat diet on young adult rats have examined multiple systemic markers of diabetes, cognitive impairments after consumption of this diet, and impairments of CA1 hippocampal pyramidal neuron intrinsic excitability as well as altered insulin sensitivity. To our knowledge, findings from our laboratory are the first evidence of broadly dichotomous effects of a high-fat diet on young reproductively intact males and female (see also [1]).

Neuroscientific publications have a pronounced sex bias in the selection of research subjects (5.5 papers use males for every 1 using females, [72]). This bias persists, despite updates in NIH guidelines and compelling evidence that females can be studied irrespective of estrous cycle state without significant increases in outcome variance [72–74]. Consistently small and similar variance in our data from males and females, across a wide range of physiological measures, argues (as detailed by McCarthy et al. [75]) that the estrous cycle stage is not a relevant variable in the sex differences seen here after 12 weeks feeding of the HFD. Mechanisms accounting for up-regulation of circulating insulin in young HFD males, and massive down-regulation of circulating insulin in young HFD females, remain to be determined, as are the cellular mechanisms driving loss (in males) and increases (in females) of CA1-neuron insulin sensitivity.

Sex differences in the development of type-2 diabetes can be profound in humans and in animal models but in humans are most studied in older populations. Studies in younger populations are rare, despite alarming human population trends of incidence of type-2 diabetes in youth [52]. Obese women with type-2 diabetes have a higher occurrence of cognitive decline than men [58] as they age. Obese women are twice as likely to have dementia as women of normal weight, while obese men are at no greater risk than normal weight men [57]. Estrogen affects insulin-degrading enzyme (IDE) activity, changing insulin degradation rates in the hippocampus and rates of catabolism of amyloid β protein [76, 77]. Our own laboratory currently is studying the sex-dependent effects of much longer term consumption of the HFD, assessing cognitive, systemic, and cellular physiological variables.

The loss of CA1 intrinsic excitability has significant cognitive consequences, over short terms and over life spans [61, 78]. Our findings indicate young females may be at particularly high cognitive risk from exposure to high-fat diets, since they are largely asymptomatic on clinical measures likely to be assessed if aberrations in diet or glucose dysregulation are suspected. Prior studies document HFD-dependent learning and memory impairments in male rats performing hippocampal-dependent tasks [50, 79, 80]. Our findings highlight an imperative for considerably more research into sex-dependent differences, relating systemic and neural plasticity mechanisms in metabolic disorders across the life span. While numerous studies of the effects of high-fat diets on a variety of outcome variables in rats have been published [26, 50, 81–83], only rarely [1, 51, 84] have direct comparisons between males and females been made. No prior studies have made the full range of direct comparisons of neuronal, cognitive, and metabolic effects between males and females as those reported here.

As seen here and in our earlier paper [1], the consequences of ingesting a high-fat diet can differ significantly between males and females, with large differences in circulating insulin and insulin sensitivity (both peripheral and central) as a result. However, both males and females exhibited similar impairments in hippocampal intrinsic excitability and consequent severe impairments in spatial memory, despite these differences. Similarly, while the mechanisms of cognitive impairment in human diabetics have not been well defined, it has been established that both type-1 and type-2 diabetics exhibit these impairments, despite strikingly different patterns of insulin secretion and insulin sensitivity ([1, 85, 86] and Figs. 6 and 7). A potential mechanism contributing to cognitive dysfunction in both insulin-insensitive and insulin-insufficient disorders may be significant sustained glucocorticoid elevation [87], a sex-independent finding we previously reported in this model (200–300 % elevations in corticosterone were observed in both HFD females and males) [1]. Finally, as reported here, CA1 pyramidal neurons from both male and female HFD rats exhibited significantly reduced intrinsic excitability, a hallmark typical of age-associated cognitive impairment [26, 29, 43–47]. Further work assessing not only insulin but also many other glucose- and lipid-regulation pathways in males *and* females to detail their mechanism for altering neuronal excitability and cognitive function is clearly needed.

Glucose and/or insulin dysregulation can play a significant role in dementia-related cognitive impairments. Intrahippocampal amyloid β_1–42_ oligomers impair both spatial working memory and hippocampal metabolism (task-associated reductions in extracellular glucose are lost), with reduced Akt phosphorylation contributing to dysfunctional insulin signaling [88]. Loss of IGF-1 and insulin receptors, and alterations in their signaling proteins IRS-1 and IRS-2, is found in Alzheimer’s patients [89]. In Alzheimer’s, neurons can become both insulin- and IGF-1-resistant, even absent the clinical diagnosis of diabetes, with elevations in IRS-1 serving as a biomarker for brain insulin-resistance [90]. Learning and memory impairments in type-2 diabetics correlate strongly with both duration of the disease [91] and subjects’ age [56]. The rate of cognitive decline associated with diabetes is also increased by the presence of the apoE4 allele [92], reinforcing emerging hypotheses linking neuronal insulin-signaling dysregulation and Alzheimer’s. Indeed, longitudinal studies demonstrate diabetics are at increased risk of developing all forms of dementia [54, 55].

Pyramidal neuron intrinsic excitability is transiently modulated in many different learning and memory tasks [12–15, 33, 37, 40, 93], including spatial memory [16, 47]. AHP modulation is significantly impaired in aging cognitively impaired models [34, 45, 47, 59]. As observed here, both male and female CA1 neurons from HFD-fed rats are excitability-impaired, with profiles resembling impairments seen in senescence. These effects likely contribute to cognitive impairments in diabetic humans and in diabetic models. Long-term consequences of obesity, glucose dysregulation, and consequent neuronal dysfunction must be studied in parallel in males and females, since a one-size-fits-all approach cannot adequately explain the experimental observations reported.

## Additional file

Additional file 1: Figure S1.
**Dose-response testing in hippocampal slices prepared from CD males was used to determine an optimal dose of insulin to assess insulin sensitivity of CA1 pyramidal neurons. A**. AHP peak amplitudes were significantly reduced by bath application of 12.5 nM insulin. **B**. AHP duration was significantly reduced by concentrations from 12.5 and 25 nM. **C**. Concentrations of 12.5 nM insulin induced significant reductions in mAHP and sAHP components, without inducing spontaneous action potential firing or other signs of instability when assessed for periods up to 45 min in vitro. ***p* < 0.01; ****p* < 0.001.
