# Supplementary figures and images for: High-fat diet impairs spatial memory and hippocampal intrinsic excitability and sex-dependently alters circulating insulin and hippocampal insulin sensitivity
Source: Biol Sex Differ. 2016 Jan 28;7:9. doi: 10.1186/s13293-016-0060-3 (PMC4730722; doi:10.1186/s13293-016-0060-3)

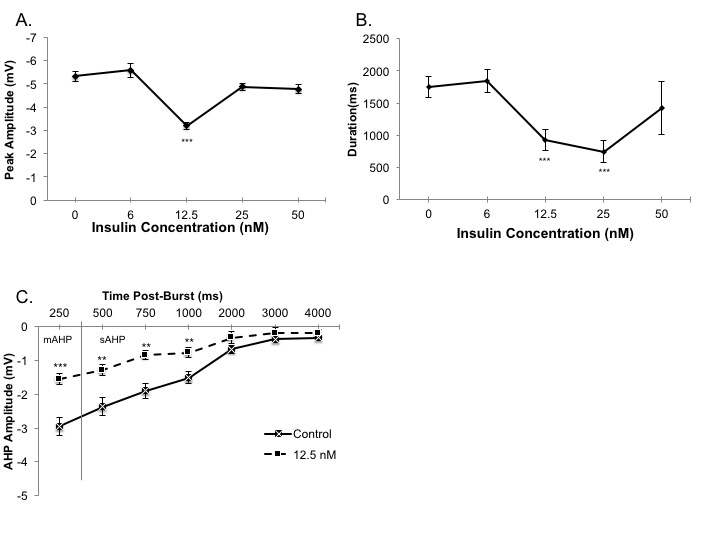

Supplement: Additional file 1: Figure S1. — Dose-response testing in hippocampal slices prepared from CD males was used to determine an optimal dose of insulin to assess insulin sensitivity of CA1 pyramidal neurons. A. AHP peak amplitudes were significantly reduced by bath application of 12.5 nM insulin. B. AHP duration was significantly reduced by concentrations from 12.5 and 25 nM. C. Concentrations of 12.5 nM insulin induced significant reductions in mAHP and sAHP components, without inducing spontaneous action potential firing or other signs of instability when assessed for periods up to 45 min in vitro. **p < 0.01; ***p < 0.001. [file 13293_2016_60_MOESM1_ESM.jpg]
